# Royal Medical and Chirurgical Society

**Published:** 1897-11-13

**Authors:** 


### ROYAL MEDICAL AND CHIRURGICAL SOCIETY.

At the meeting held on November 9th two papers were read, each of which was of considerable interest. The number of Fellows present, however, was extremely small.

### Cystic Tumour of the Bladder.

Mr. P. J. Freyer described a case which he thought to be unique in the annals of surgery. The patient, an Anglo-Indian officer, aged 36, had suffered for ten years from obscure urinary symptoms, which dated from a fall in the cricket field in 1886, by which he hurt his loins. The injury was followed by pain in the region of the right kidney, intermittent hæmaturia, pyrexia, and cystitis.

The most prominent symptom for several years latterly had been that the patient could never empty his bladder in the natural way. With much straining he could pass two or three ounces of water, when the stream stopped, and he could only empty his bladder by passing a catheter or by lying down. High fever accompanied by much pus in the urine periodically occurred. The patient was extremely thin, weak, and depressed from long suffering. He had been repeatedly sounded by various surgeons, but no stone had been found.

The patient was first seen on September 24th last, and was then carefully sounded, but no stone was found. Mr. Freyer suspected the presence of a tumour of the bladder which obstructed the flow of urine by falling against the neck of that viscus. The cystoscope confirmed these suspicions, at once revealing a smooth tumour the size of a walnut, with thick rounded pedicle, growing from the right side of the base of the bladder.

On September 30th the bladder was opened suprapubically, and on introducing the finger the tumour was found, being the size of a walnut, smooth on the surface, except at the apex, where a small papilloma was growing from it, slightly bilobed, and attached by a round tense pedicle to the bladder wall near the right ureteral opening. On twisting off a portion by the forceps and then drawing the tumour into the abdominal wound it was found that it was cystic, and contained two uric calculi—one the size and shape of a nutmeg, the other the size of a large pea, the two weighing 42 grains. The tumour had been rendered tense by the calculi and contained urine, but was now lying flaccid, like the finger of a glove, inside the bladder. The pedicle was clamped, the tumour was excised, and the pedicle was then cauterised. Then the pedicle was transfixed, and tied in two parts by silk ligatures, the ends of which were taken out through the abdominal wound. Perfect recovery ensued, the ligatures came away on the tenth and twelfth days respectively, and the wound completely closed within a month. The patient had had no urinary symptoms since, had put on flesh, and was in perfect health, playing hockey in public matches.

The author considered that the tumour was formed by the calculi passing down from the kidney as far as that portion of the ureter which passes obliquely through the bladder wall, stopping short of the ureteral opening; that, impelled onwards by the force of the urine, they bulged the walls of the bladder and ureter inwards, and that in this way eventually a pendulous cystic tumour, containing the calculi, and communicating by a narrow opening with the ureter, was formed.

